# Pseudomonas Aeruginosa Lectins As Targets for Novel Antibacterials

**Published:** 2015

**Authors:** A. V. Grishin, M. S. Krivozubov, A. S. Karyagina, A. L. Gintsburg

**Affiliations:** Gamaleya Research Center of Epidemiology and Microbiology, Gamaleya Str., 18, Moscow, 123098, Russia; Institute of Agricultural Biotechnology, Timiryazevskaya Str., 42, Moscow, 127550, Russia; Belozersky Institute of Physical and Chemical Biology, Lomonosov Moscow State University, Leninskie Gory, 1, bld. 40, Moscow, 119991, Russia

**Keywords:** Pseudomonas aeruginosa, lectin, LecA, LecB, antibiotic resistance, biofilm, inhibitor

## Abstract

*Pseudomonas aeruginosa *is one of the most widespread and troublesome opportunistic pathogens that is capable of colonizing various human tissues and organs and is often resistant to many currently used antibiotics. This resistance is caused by different factors, including the acquisition of specific resistance genes, intrinsic capability to diminish antibiotic penetration into the bacterial cell, and the ability to form biofilms. This situation has prompted the development of novel compounds differing in their mechanism of action from traditional antibiotics that suppress the growth of microorganisms or directly kill bacteria. Instead, these new compounds should decrease the pathogens’ ability to colonize and damage human tissues by inhibiting the virulence factors and biofilm formation. The lectins LecA and LecB that bind galactose and fucose, as well as oligo- and polysaccharides containing these sugars, are among the most thoroughly-studied targets for such novel antibacterials. In this review, we summarize the results of experiments highlighting the importance of these proteins for *P. aeruginosa* pathogenicity and provide information on existing lectins inhibitors and their effectiveness in various experimental models. Particular attention is paid to the effects of lectins inhibition in animal models of infection and in clinical practice. We argue that lectins inhibition is a perspective approach to combating *P. aeruginosa*. However, despite the existence of highly effective *in vitro *inhibitors, further experiments are required in order to advance these inhibitors into pre-clinical studies.

## INTRODUCTION

*Pseudomonas aeruginosa *is a widespread bacterium that can have both saprotrophic and parasitic lifestyles. It can colonize virtually every human tissue and cause a number of acute and chronic diseases, including acute pneumonia, bacteriemia, urinary tract infection, external otitis, dermatitis, wound and burn sepsis, keratitis, meningitis, brain abscess, endocarditis, and various bone and joint infections. *P. aeruginosa *is an opportunistic pathogen; it typically affects people with weakened immune systems, being one of the most problematic hospital-acquired pathogens.. According to current data, at least 10–15% of all hospital-acquired infections are caused by *P. aeruginosa* [1, 2]. Furthermore, *P. aeruginosa *often colonizes the lungs of patients with cystic fibrosis, the hereditary disease associated with insufficient chloride canal function and mucus accumulation in lungs, reducing the lung function and the patient’s life expectancy [2].

One of the main challenges associated with the therapy of *P. aeruginosa *infections is that the pathogen shows resistance to many antibiotics. Its resistance to antibiotics consists of several aspects. First, the pathogen controls the level of porins and membrane permeability for antibacterials and expresses a large number of efflux pumps involved in the excretion of antibiotic molecules from the cell. Second, *P. aeruginosa*, similar to many other pathogens, can easily acquire specific antibiotic resistance genes (e.g., the genes coding for β-lactamases and aminoglycoside-inactivating enzymes) [2]. Finally, chronic infections caused by *P. aeruginosa* are accompanied by biofilm formation. Biofilms are organized microbial communities submerged into the extracellular polymer matrix, which consists of polysaccharides, proteins, and DNA synthesized by these microorganisms [3, 4]. Inside biofilm, bacteria become significantly more resistant to unfavorable environmental conditions, as well as to antimicrobial agents and factors of the human immune system [3]. *P. aeruginosa* forms difficult-to-remove biofilms in patients’ organs and tissues, as well as on implanted devices and catheters [3, 5]. One of the popular approaches to solving this problem suggests the design of new substances that would either inhibit or inactivate the virulence factors of pathogenic bacteria (toxins, adhesins, effector proteins modulating the metabolism and the immune response of the host organism, secretion systems delivering these proteins to the target site, and factors facilitating communication between the bacteria and biofilm formation) rather than kill the pathogens by inhibiting their biosynthesis [6]. In other words, the strategy consists in disarming rather than killing the pathogen. Resistance to these antivirulent compounds is expected to develop in slower fashion, since they will not have a direct effect on bacterial viability but will only affect their ability to infect humans.

The *P. aeruginosa *lectins LecA and LecB are viewed as potential targets for such antiviral compounds. They are soluble proteins binding galactose (LecA) and fucose (LecB) residues both individually and within oligo- and polysaccharides. These proteins are believed to be involved in the attachment of the pathogen to human cells, to be capable of epithelial tissue damage, and to play a crucial role in the formation of *P. aeruginosa* biofilms, thus acting as key virulence factors. In this review, we have summarized the results of studies focused on the role of lectins LecA and LecB in the pathogenesis and formation of biofilms, described currently known inhibitors of these proteins, and assessed the potential for using these proteins as targets to treat infections caused by *P. aeruginosa.*

## P. AERUGINOSA LECTINS: GENERAL INFORMATION

**Fig. 1:**
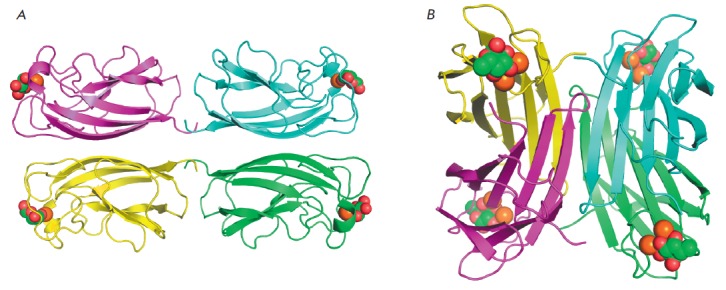
General view of LecA (A) and LecB (B) tetramers. Individual monomers are shown as polypeptide chain trace models of different colors, where flat arrows indicate β-strands. Calcium ions are shown as orange spheres, and lectinbound galactose and fucose are shown as green (carbon) and red (oxygen) spheres

Lectins LecA and LecB (also commonly known as PA-IL and PA-IIL) were isolated from *P. aeruginosa* in the 1970s as proteins capable of agglutinating human and animal erythrocytes [7-9]. Both lectins are small proteins 121 (LecA) and 115 (LecB) amino acid residues in size (12.8 and 11.9 kDa, respectively) [10, 11]. LecA binds *D*-galactose and, with lower affinity, N-acetyl-D-galactosamine. *L*-fucose exhibits the highest affinity to LecB, but this lectin also binds mannose and a number of other saccharides. Although LecA and LecB have completely different amino acid sequences, their quaternary structures are similar: both lectins form homotetrameric complexes where each monomer has its own ligand-binding site. Thus, a single tetramer can bind four molecules of the corresponding carbohydrate [12, 13] (*Fig. 1*). In *Pseudomonas *genus, the *lecA* and *lecB *genes are unique to *P. aeruginosa*; however, homologs are found in such bacteria as *Burkholderia* and *Photorhabdus*.

Regulation of lectins synthesis (mostly for LecA) has been studied rather thoroughly. Synthesis of both lectins is induced when the bacterial culture reaches a stationary phase and is regulated by *rhl *and the Pseudomonas quinolone signal (PQS), components of the quorum sensing system [14, 15]. The function of this system is based on the release of low-molecular-weight substances of different natures by these bacteria (in particular, acyl homoserine lactones and quinolones), which allows them to send signals about their presence to other bacteria. That is how bacteria “feel” that a certain population density level has been reached and trigger the expression of virulence factors (such as LasA and LasB proteases, exotoxin A, alkaline protease, etc.) and biofilm formation [16]. The regulation of LecA expression is very similar to the regulation of the synthesis of pyocyanin, an important toxin inducing oxidative stress and damage to the cells of the host organism [14, 15, 17-20]. Interestingly, the level of produced LecA, as well as other virulence factors, increases when the pathogen comes into contact with certain molecules produced by the host organism under stress conditions: noradrenalin, IFN-γ, adenosine, and κ-opioid peptide dynorphin [19, 21-23].

*P. aeruginosa *lectins are mostly localized in the cell cytoplasm; a certain amount of them can be found on the outer membrane surface [24, 25]. LecB on the outer membrane surface is most likely bound to fucose residues of glycolipids or glycoproteins [25, 26]. It has been demonstrated that LecB interacts with one of the main outer membrane porins of *P. aeruginosa* OprF and is not detected on the membrane of bacteria with mutations in the *oprF *gene [26]. However, taking into account the fact that these mutations significantly change the overall properties of the *P. aeruginosa* outer membrane [27], it is not inconceivable that other proteins can also be involved in anchoring LecB to the membrane. As opposed to LecB, localization of LecA remains virtually unstudied.

## ROLE OF LECTINS IN PATHOGENESIS

**Fig. 2:**
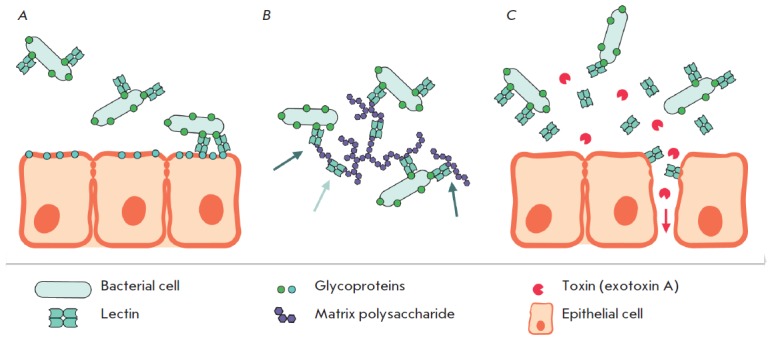
Proposed functions of *P. aeruginosa *lectins: adhesion to host epithelial cells (A); attachment of bacterial cells to biofilm matrix polysaccharides and cross-linking of these polysaccharides (B); disruption of epithelial barrier function and increase in permeability for other virulence factors (C). Light gray arrow indicates cross-linked polysaccharides, dark gray arrows indicate polysaccharides attached to bacteria, red arrow depicts toxins permeation through the disrupted epithelium

The role of lectins LecA and LecB in the pathogenesis of the diseases accompanying a *P. aeruginosa *infection is not yet unambiguously determined. Some data demonstrate that these lectins enhance adhesion of the bacteria to the substrate (e.g., human cells), are involved in the aggregation of bacterial cells, biofilm formation, and interaction between a bacterium and the host organism’s tissues, resulting in tissue damage. The presumed role of lectins has been schematically summarized in *Fig. 2*.

**Adhesion**

LecA and LecB lectins bind the oligosaccharides of many human and mammalian glycoproteins [28-34], thus naturally suggesting that lectins are directly involved in the adhesion of *P. aeruginosa *to human tissues [35]. Adhesion is a crucial stage in pathogenesis. Adhesion of bacterial cells to the epithelial tissue surface precedes colonization, which may subsequently lead to biofilm formation or pathogen invasion. However, the experimental data on the role of LecA and LecB lectins in *P. aeruginosa *adhesion are controversial. Wentworth *et al*. [36] studied bacterial adhesion to a rabbit corneal epithelium culture. Addition of bacterial cell lysate was shown to increase the amount of adhered intact bacteria; this effect was partially inhibited by addition of galactose, mannose, and fucose. A conclusion was drawn that stimulation of adhesion is associated with the release of lectins from the cytoplasm of the lysed bacterial cells. Binding of bacteria to fibronectin, one of the most common human glycoproteins, was also inhibited by addition of saccharides: to the greatest extent, by adding sialic acid, N-acetylglucosamine and N-acetylgalactosamine and to a lower extent, by adding galactose and fucose [37]. However, as opposed to the previous study, addition of LecA did not increase but reduced the amount of bacterial cells bound to immobilized fibronectin. *P. aeruginosa *strains with mutations in the *lecA *and *lecB *genes retain their ability to bind to mucins (glycoproteins secreted by epithelial cells) [38], while their ability to bind to A549 human lung epithelial cells significantly deteriorates [39, 40]. It was also demonstrated that binding to A549 cells is inhibited in a dose-dependent manner by lectin ligands, methyl-β-galactoside and methyl-α-fucoside [40], while binding to immortalized human airway epithelial cells NuLi (derived from a healthy donor) and CuFi (derived from a cystic fibrosis patient) is inhibited by the addition of anti-LecB antibodies, but not control non-specific antibodies [41]. Contrariwise, Eierhoff *et al. *[42] demonstrated that interaction between lectin LecA and globotriaosilceramide (Gb3) is required for the invasion of *P. aeruginosa *inside H1299 human lung epithelial cells and inside artificial vesicles but plays no role in adhesion. Bacteria with mutation in the *lecA* gene bind to H1299 cells and artificial vesicles with the same efficiency as wild-type bacteria do. These contradictions probably arose from the fact that different substrates were used to study adhesion (isolated glycoproteins, epithelial cells of different origin) and on whether lectins had an effect on the binding to a certain substrate or did not depend on the range of oligosaccharides present on the substrate surface.

It should also be mentioned that in addition to lectins,* P. aeruginosa *adhesion to the cells of a host organism is also ensured by other factors, such as flagella and type IV pili [43, 44]. It is rather difficult to distinguish between the effects arising from the presence of different adhesins. Furthermore, it is known that functional LecB is required to ensure normal assembly of* P. aeruginosa *pili and secretion of certain proteins [38]. Hence, although *P. aeruginosa *lectins play a crucial role in binding the pathogen to certain types of human cells, the mechanism underlying this process has not been fully elucidated; its role in *in vivo *infection remains uncertain; and the contribution of lectins in it can be either direct (interaction with glycan structures on the cell surface) or indirect (involvement in assembly, secretion, and functioning of other adhesins, such as type IV pili).

**Biofilms**

Both lectins are involved not only in adhesion, but also in the formation of *P. aeruginosa *biofilms. Independent research groups have used different experimental models to demonstrate that *P. aeruginosa *strains with mutations in the *lecA *and *lecB *genes form poorly developed biofilms without the well-defined architecture that is typical of the biofilms of wild-type strains [25, 38, 45, 46]. Furthermore, addition of isopropyl-β-* D*-thiogalactopyranoside (IPTG) or nitrophenylgalactoside (galactose derivatives capable of binding to LecA with a higher affinity than galactose) when *P. aeruginosa* biofilms were grown on steel coupons inhibited biofilm formation to the level of the *lecA *mutant (the surface area of the biofilm was twice as low compared to that of wild-type biofilms grown without IPTG), while addition of galactosides to the already formed biofilms resulted in their dispersal. It is noteworthy that galactosides affected neither formation nor dispersal of the biofilm formed by the strain with a mutation in the *lecA *gene [45]. Identically, biofilms formed by *P. aeruginosa *with mutations in the *lecB *gene on cover slips were much thinner and had a smaller surface area than the wild-type biofilms [25, 46]. Similar to galactosides, LecB ligand nitrophenylfucoside prevented biofilm formation and partially dispersed wild-type biofilms but not those of *lecB *mutant. It is an interesting fact that nitrophenylfucoside inhibited biofilm formation not only by the laboratory strain PAO1, but also by three clinical isolates [46].

Unfortunately, although these studies demonstrate that functional lectins genes are needed for the formation of full-fledged biofilms, the direct function of lectins in this process remains unclear. The role of lectins may possibly be associated with the aggregation of bacterial cells and microcolony formation. At least Diggle *et al*. detected no microcolony formation by the* lecA *mutant [45]. LecB lectin is needed for proper assembly of type IV pili, which, in turn, are required for biofilm formation [38]. Lectins may potentially facilitate binding of polysaccharides of the biofilm extracellular matrix to bacterial cells or are required to cross-link individual chains of these polysaccharides. Cross-linking polysaccharide chains by multivalent lectins can potentially facilitate the formation of denser biofilms that would be more resistant to physical impact. Interestingly, the extracellular polysaccharide Psl that is absolutely required for the formation of *P. aeruginosa *biofilms contains mannose and, according to some sources, galactose, which are ligands of lectins LecB and LecA, respectively [47, 48]. Binding of this polysaccharide to bacterial cells is required to initiate the biofilm formation process [49].

**Effect on epithelial cells**

The direct effect of lectins on human airway and intestinal epithelial cells was investigated in several studies. It has been demonstrated that addition of LecA significantly slows the growth of nasal polyp epithelial cells and reduces the number of ciliated cells. Furthermore, LecA causes formation of large vacuoles in the cells and, when added at large concentrations, even cell detachment [50]. Incubation with LecA also significantly reduces the ciliary beat frequency [51, 52]. The effect of LecA on the ciliary beat frequency was attenuated by adding *D*-galactose. Ciliary beat was inhibited by LecB, and this effect was attenuated by adding fucose [51-54]. In the norm, movements of airway epithelium ciliated cells facilitate the removal of mucus and foreign particles trapped by it (including bacterial cells) from the lungs. Inhibition of the ciliary function is most likely to be caused by binding of lectins to glycoproteins on the surface of epithelial cells and the response of epithelial cells to this event or directly by cilia cross-linking to one another [51]. However, these effects have been demonstrated only in *in vitro *models and it remains unclear how important they are in an airway infection *in vivo*.

LecA lectin has a negative effect on intestinal epithelium. In particular, addition of LecA to Caco-2 and T-84 cell cultures significantly reduces the transepithelial electrical resistance of the cellular monolayer and increases monolayer permeability for mannite; this effect is attenuated by N-acetylgalactosamine [21, 55, 56]. The most likely reason is that lectin disrupts tight intercellular contacts [55]. Increased permeability of intestinal epithelium was observed *in vivo *using a mouse model of intestinal infection [21, 55, 56]. The fatality rate 48 h after LecA, in combination with exotoxin A or elastase, was injected into the cecum of mice previously subjected to 30% partial hepatectomy was 100%. This effect was not observed when LecA, exotoxin A, or elastase was injected as an individual substance. Injection of the clinical isolate of *P. aeruginosa *(but not the mutant incapable of LecA expression) caused a 100% mortality rate. Taking into account that intravenous injection of endotoxin A is fatal to mice, it is most likely that injection of LecA into the cecum renders epithelium permeable to endotoxin A, which enters the blood flow.

## LECTIN LIGANDS AND INHIBITORS

Lectins perform their functions by binding oligo- and polysaccarides, whether they are human or bacterial glycoprotein oligosaccharides or matrix polysaccharides of *P. aeruginosa *biofilms. Specificity of lectins with respect to the saccharides being bound plays the key role in this process.

**Fig. 3:**
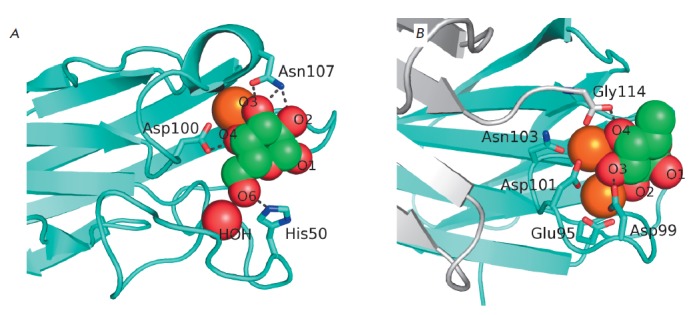
Detailed view of LecA (A) and LecB (B) sugar-binding sites. Lectins are shown as polypeptide chain trace models, where flat arrows indicate β-strands. Calcium ions are shown as orange spheres, and lectin-bound galactose and fucose are shown as green (carbon) and red (oxygen) spheres. The water molecule involved in galactose binding by LecA is shown as a red sphere, and the side chains of certain amino acid residues involved in sugar binding or calcium coordination are shown as sticks. Black dotted lines depict hydrogen bonds between the sugars and side chains of amino acid residues. The additional monomer of LecB is shown in gray (B); C-terminal glycine of this monomer is involved in the formation of the sugar-binding site of the neighboring monomer

Lectin LecA preferentially binds to α-*D*-galactose and oligo- and polysaccharides containing terminal non-reducing residues of α-*D*-galactose, such as the B, P^k^ and P_1_ blood group antigens, melibiose and galactobiose, plant-derived galactomannans, etc. [28-30, 57]. Similar to many other lectins, the galactose-binding site of LecA contains a calcium ion bound to protein carboxyl groups via coordination bonds. The galactose molecule is present in the binding site in its most stable ^4^C_1_ conformation; the O_3_ and O_4_ atoms are involved in coordination bonds with an immobilized calcium ion; the O_2_, O_3_, and O_4_ atoms form additional hydrogen bonds with the amino acid residues of the protein; and the O6 atom forms hydrogen bonds with a water molecule that is firmly fixed by two hydrogen bonds in the binding site [12, 58] (*Fig. 3A*). The dissociation constant of the LecA–galactose complex is 88 µM [59]. The terminal residue of α-*D*-galactose plays the key role in binding of oligosaccharides by lectin LecA, while other oligosaccharide residues form few contacts with the protein [31, 58]. In this connection, the oligosaccharide affinity to LecA may vary within a rather narrow range depending on the composition of the oligosaccharide and the details of the glycoside bond connecting the terminal galactose residue to the next residue in the oligosaccharide: the dissociation constants typically vary from 30 to 130 µKM [31, 58]. In addition to α-*D*-galactose, LecA can also bind N-acetyl-*D*-galactosamine (although with lower affinity) [7, 60], as well as adenine and acyl-homoserine lactones; however, an independent binding site is involved in the latter interaction [61, 62].

LecB has a broader specificity and higher affinity to its ligands. It can bind *L*-fucose and *L*-fucosylamine,* L*-galactose, *D*-arabinose, *D*-mannose, and *D*-fructose [63-65]. The dissociation constant of the LecB–*L*-fucose complex is 2.9 µKM; its interaction with other saccharides is weaker [65]. The reason for such high affinity for lectin is that there are two immobilized calcium ions in the LecB ligand-binding site; coordination interactions with these ions determine binding between saccharides and LecB (*Fig. 3B*). The optimal arrangement of the saccharide hydroxyl groups for coordinating two calcium ions by LecB corresponds to two hydroxyl groups in the equatorial position and one hydroxyl group in the axial position. This fact makes a landmark contribution to LecB specificity: all the saccharides bound by LecB have this arrangement of hydroxyl groups in their most energetically favorable conformations [63, 65]. Like LecA, LecB interacts with oligosaccharides having terminal non-reducing residues of the corresponding monosaccharides, in particular *L*-fucose. It has been demonstrated that LecB can bind oligosaccharides of the A, B, H, Le^a^ and Le^x^ blood groups [28, 32, 66, 67]. The terminal fucose residue makes the main contribution to the energy of interaction between the oligosaccharides and LecB, although the oligosaccharide affinities can be increased 14-fold compared to that of fucose due to the composition and positions of other monosaccharide residues, which has been demonstrated for Le^a^ [32].

A large number of various LecA and LecB inhibitors have been proposed over the past decade. Except for glycomimetic peptides [52], all of them contain residues of the corresponding saccharides as affine groups. These inhibitors include monosaccharide derivatives, multivalent glycoclusters and dendrimers of different chemical nature, and natural glycoproteins and polysaccharides.

**Monovalent monosaccharide derivatives**

Many monosaccharide derivatives bind to *P. aeruginosa* lectins with a higher affinity than the original saccharides do. For example, even small hydrophobic substituents at the first oxygen atom increase the affinity of the corresponding saccharides both to LecA and LecB. The dissociation constant of the LecA–IPTG complex is almost threefold lower than that of the LecA–* D*-galactose complex [59, 60], while the dissociation constant of the LecB–methyl-α-*L*-fucoside complex is sevenfold lower than that of the LecB complex with unmodified *L*-fucose [65].

**Fig. 4:**
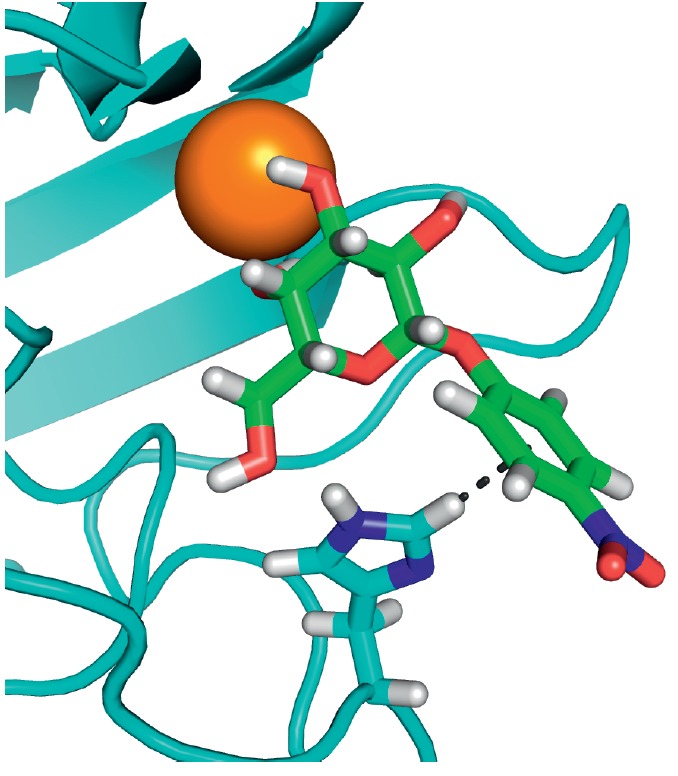
LecA – nitrophenylgalactoside complex. Nitrophenyl- galactoside and side chain of LecA His50 are shown as sticks, and the black dotted line depicts CH-π interaction

The affinity of galactosides to LecA can be further increased by inserting simple aromatic substituents. *K*_d_ of the complex between LecA and such compounds as phenylgalactopyranoside, *p*-nitrophenylgalactopyranoside (*Table*, compound 1) ,*p*-aminophenylgalactopyranoside,* p*-tolylgalactopyranoside, naphthylgalactopyranoside, etc. is 4–15 µM (let us remember that the* K*_d_ of unsubstituted D-galactose is almost 90 µKM) [68]. This is associated with the formation of a contact between the hydrogen atom and the ε-carbon atom of LecA His50 and the aromatic ring π-system (*Fig. 4*). This interaction is known as the CH-π interaction, and its energy is ~ 1 kcal/mol. For the sake of comparison, the energy of interaction between LecA and *D*-galactose is 6.0 kcal/mol. The mechanism of this interaction is similar to that of the hydrogen bond; however, it is not an electronegative atom with an unshared electron pair that acts as a hydrogen acceptor but the aromatic π-electron system [68]. Insertion of aliphatic substituents or aromatic ones separated from galactose by analiphatic linker preventing the formation of an CH-π interaction provides for a much lower increase in affinity [68-71].

**Table:** Some of the most effective inhibitors of P. aeruginosa lectins

No.	Chemical formula of the matrix (for multivalent compounds)	Chemical formula of the functional group	Lectin target, reference	Valence	Affinity (ITC: K_d_; ELLA: IC_50_)	Improvement of affinity as compared to monosaccharide (calculated per monosaccharide – shown in parentheses)
1		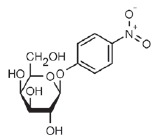	LecA, [59]	1	ITC: 14.1 µM	ITC: 6 (6)
2		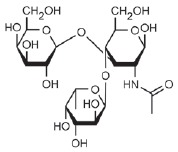	LecB, [32]	1	ELLA: 0.51 µM; ITC: 0.2 µM	ELLA: 12 (12) ITC: 14 (14)
3	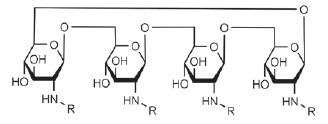	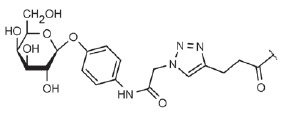	LecA, [71]	4	ELLA: 57 nM; ITC: 79 nM	ELLA: 1210 (300) ITC: 1114 (278)
4	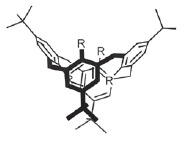	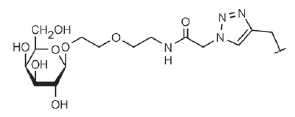	LecA, [70]	4	ELLA: 7 µM; SPR: 1.0 µM; ITC: 90 nM	ELLA: 50 (12) SPR: 58 (14) ITC: 978 (244)
5		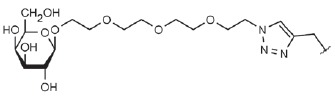	LecA, [40,80]	4	SPR: 500 nM ITC: 176 nM	SPR: 143 (35) ITC: 500 (125)
6		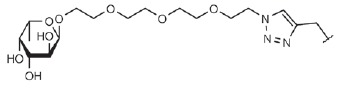	LecB, [40]	4	ITC: 48 nM	ITC: 60 (15)
7	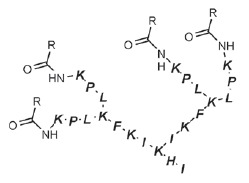	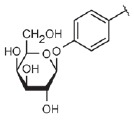	LecA, [59]	4	ITC: 0.1 µM	ITC: 880 (220)
8		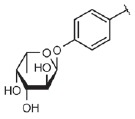	LecB, [46]	4	ELLA: 0.14 µM	ELLA: 79 (20)
9	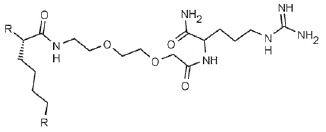	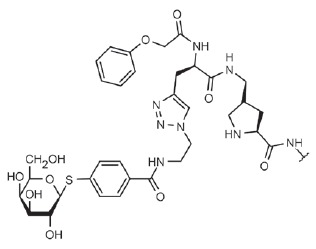	LecA, [91]	2	ITC: 82 nM	ITC: 1073 (537)
10	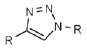	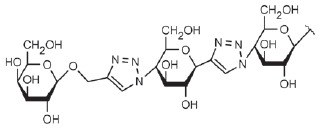	LecA, [89]	2	Inhibition of FITC-LecA binding, IC_50_: 2.7 nM; ITC: 28 nM	Inhibition of FITC-LecA binding: 7407 (3703) ITC: 3143 (1572)
11	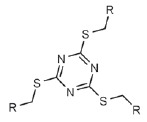	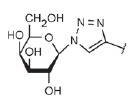	LecA, [79]	3	ITC: 1.1 µM	ITC: 80 (27)
12		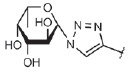	LecB, [79]	3	ITC: 50 µM	ITC: 0.6 (0.2)

Lectin LecB does not exhibit such a simple dependence. Among monovalent ligands, oligosaccharide Le^a^ (*Table*, compound 2) has the greatest affinity to LecB [32]. The *K*_d_ of the LecB–Lea complex is 210 nM, while that of the LecB–fucose complex is 2.9 µM. Although several attempts have been made to design ligands that would exhibit higher affinity than Lea, this objective has not been achieved. Various derivatives of disaccharide α-*L*-Fuc-(1→4)-β-*D*-GlcNAc – the component of Le^a^ – have been characterized by an affinity to LecB identical to that of Le^a^. An analysis of the crystalline structure of the complex has demonstrated that the inserted substituents are not involved in the formation of contacts with the protein [72]. A series of fucosylamides have been designed where the first oxygen atom of fucose is replaced with a nitrogen atom of the amide group carrying different appreciably bulky substituents of non-saccharide nature [73]. All the compounds were bound to LecB with dissociation constants of 0.68–2.1 µM, which shows no improvement compared to Lea and even methylfucoside. The reason is that the amide group cannot interact with the conservative water molecule that participates in the ligand binding to lectin LecB. Finally, Hauck *et al*. [74] have designed several classes of derivatives of methyl-*D*-mannoside, another saccharide that binds to LecB with an affinity lower than that of LecB binding to fucose (K_d_ = 71 µKM). Some amide and sulfonamide derivatives at the 6^th^ position of mannose show a significant increase in affinity. For example, *K*_d_ for one of sulfonamides is 3.3 µKM. Although this value is 20-fold higher compared to the initial methylmannoside, no improvement compared to fucose and Le^a^ was achieved.

Thus, there are no monovalent ligands with an affinity to lectins higher than that of unmodified monosaccharides by more than an order of magnitude. This is one of the reasons why researchers have focused on designing multivalent inhibitors.

**Multivalent compounds**

Lectins, irrespective of their origin (plant, animal, or bacterial), typically bind saccharides with an appreciably low affinity [75]. This limitation can be overcome through the multivalence of both lectins and their ligands. Multivalence implies that a single molecule or the molecular complex contains several identical binding sites. For example, lectins can be organized into homomultimeric protein complexes, while glycoproteins (lectin receptors) can carry several identical glycan chains bound by lectins. This multivalence allows one to significantly increase the affinity and specificity of the interaction between lectins and glycans via several mechanisms. First, in some cases several sites of multivalent lectin can simultaneously bind several epitopes of a multivalent ligand. Such interaction is known as a chelate or bridging interaction. Second, even if simultaneous binding is impossible, the presence of several epitopes that can interact with lectin on a single ligand molecule increases the local concentration of these epitopes. During dissociation of the lectin complex with a single epitope, lectin has a high probability of binding to another identical epitope that is located nearby. This mechanism of ligand entrapment is known as statistical rebinding [76, 77]. These effects have recently been used increasingly often to design multivalent compounds inhibiting the effect of lectins: glycoclusters, glycodendrimers, and glycopolymers [76, 78].

*P. aeruginosa *lectins were no exception. A large number of compounds belonging to different chemical classes, with different valences and different linkers between the saccharide and the core of the multivalent compound, have been designed for both lectins of this pathogen (in particular, for LecA). Glycoclusters based on trithiocyanuric acid [79], calixarenes, and resorcinarenes [40, 70, 80-82]; linear and cyclic β-peptoids, porphyrin [81], fullerenes [83], and cyclooligosaccharides [71]; polyphenylacetylene polymers functionalized with galactose residues and gold nanoparticles [85]; dendrimers of peptide [59, 86] and non-peptide nature [87, 88]; and bivalent compounds [89-91] were proposed for use as multivalent inhibitors of LecA. The LecB inhibitors included synthetic oligomers based on pentaerythrityl phosphodiester [92], dendrimers based on lysins and cyclopeptides [93], those based on *D*- and* L*-oligopeptides [46, 94, 95], glycoclusters based on trithiocyanuric acid [79] and calixarenes [40], as well as biand trivalent compounds functionalized by disaccharide α-*L*-Fuc-(1→4)-β-*D*-GlcNAc instead of fucose [96].

Many of these compounds are characterized by a significantly increased affinity to the corresponding lectin in isothermal titration calorimetry (ITC) experiments and efficiency in inhibition of lectin binding to immobilized saccharides in enzyme-linked lectin assay (ELLA). For example, the *K*_d_ values of the complexes of LecA with galactosylated glycoclusters based on cyclic oligosaccharides (*Table*, compound 3) [71] and calixarenes (*Table*, compound 4) [70], oligopeptide dendrimer (*Table*, compound 7) [59], and the bivalent ligand selected during screening of a library consisting of 625 compounds (*Table*, compound 9) [91] were ~ 80–100 nM, which is almost 1,000-fold lower than the *K*_d_ of galactose. The bivalent ligand where two galactose residues are connected by a rigid linker ~24 A long is characterized by the highest affinity to LecA; its *K*_d_ is 28 nM (*Table*, compound 10) [90]. According to the molecular modeling data, all these ligands can bind two monomers of a LecA tetramer, thus providing a chelate effect, which is probably responsible for such a significant increase in affinity. In the LecB tetramer, the distance between the fucose-binding sites in the adjacent monomers is much greater (~26–28 A in LecA and at least 35–37 A in LecB), and the increase in affinity due to the multivalence in LecB inhibitors is significantly lower.

Despite the great variety of synthesized multivalent compounds, only relatively few studies have focused on their effect on bacterial cells, adhesion, or biofilm formation. Oligopeptide dendrimers are capable of inhibiting biofilm formation. One of these dendrimers, GalAG2 (*Table*, compound 7) with four galactose residues, virtually completely inhibits the formation of *P. aeruginosa* biofilms on steel coupons and facilitates the dispersion of already formed biofilms. Unfortunately, attempts to optimize the amino acid sequence of the oligopeptide only slightly improved its ability to disperse the biofilms rather than to inhibit their growth [59, 86]. Similar fucosylated peptide dendrimers were synthesized as LecB inhibitors (*Table*, compound 8) [46, 94]. The tetravalent dendrimer FD2 effectively inhibited biofilm formation by the standard PAO1 strain and by three clinical isolates but not by the strain with a mutation in the *lecB *gene. Similar to GalAG2, it facilitated the dispersion of already formed biofilms. Tetravalent glycoclusters based on calixarenes functionalized with galactose and fucose (*Table*, compounds 5 and 6) proved also capable of inhibiting the formation of *P. aeruginosa* biofilms by PAO1 [40]. Interestingly, these glycoclusters inhibited the biofilm growth not only of the PAO1 strain, but also of the *lecA *and *lecB *gene mutants, thus demonstrating that these compounds can potentially affect other targets as well. These glycoclusters inhibited bacterial adhesion to A549 cells by 70 and 90% (glycosylated and fucosylated glycoclusters, respectively). The inhibition was more significant compared to that observed when the *lecA *or *lecB *genes were inactivated, which also suggests that these compounds may affect other targets. Trivalent glycoclusters based on trithiocyanuric acid functionalized either by galactose or by fucose (*Table*, compounds 11 and 12) are also capable of inhibiting biofilm formation. Although the affinity of these glycoclusters to the corresponding lectins is significantly lower, their effective concentrations suppressing biofilm formation are the same as those for calixarenes (5 mM). Novoa *et al*. [91] demonstrated that the bivalent LecA ligand (Table, compound 9) at a concentration of 0.05–5 µM can prevent the penetration of* P. aeruginosa *inside H1299 cells by 50–80%, although no dose dependence was revealed.

**Natural compounds**

The ability to bind *P. aeruginosa *lectins has been revealed not only in chemically synthesized but also in many natural compounds. Unfortunately, most of these compounds have not been isolated into components and only a certain degree of assumption can be made regarding the nature of their active components. Furthermore, their ability to interact with lectins has been typically demonstrated only using hemagglutination assay and western blot hybridization, without the use of more reliable quantitative procedures.

Hemagglutination assay and western blot demonstrated that the proteins of pigeon and quail egg whites [97, 98], components of honey and royal jelly [99], human breast milk and milk from some other mammals [66, 100], and extracts from the seeds of some edible plants [101] can interact with *P. aeruginosa* lectins.

Two independent research groups have demonstrated that hemagglutination induced by lectin LecA is inhibited by galactomannans, plant-derived polysaccharides consisting of linear chains of poly-(1→4)-mannose with galactose residues bound to some mannose residues via the 1→6 glycoside bond [57, 102]. Furthermore, galactomannan from guar, rather than oat glucan and some other plant-derived polysaccharides, has inhibited biofilm formation by the clinical isolate of *P. aeruginosa *[102]. The action of galactomannan, like that of peptide dendrimers, is probably based on the multivalence effect.

## IN VIVO LECTIN INHIBITION

The positive effect of inhibition of lectins LecA and LecB has been demonstrated both in *in vitro *and *in vivo *experiments: the use of lectin-specific monosaccharides and synthetic inhibitors was studied using animal models of the infection. Furthermore, single cases of using monosaccharides in clinical practice have been reported.

In the aforedescribed experiments on a model of intestinal infection in mice subjected to 30% partial hepatectomy, 107 CFU *P. aeruginosa *injected into the cecum caused 100% fatality, and so did a combination of lectin LecA and exotoxin A. However, 13% N-acetylgalactosamine added to the injection mixture reduced the fatality rate almost to zero in both cases [55]. The effect of the addition of simple saccharides (lectin ligands) was also observed in a mouse model of lung infection [39]. Intratracheal injection of *P. aeruginosa* PAO1 increased the permeability of lung epithelium and resulted in fluid accumulation in the lungs and bacterial dissemination in the organism. Infection with strains with mutations in the *lecA *or *lecB *genes had a much smaller effect on lung permeability and caused lower bacterial dissemination both in the lungs and blood of the infected mice, although their survival rates remained the same compared to mice infected with the wild-type strain. The addition of saccharides binding to LecA (N-acetylgalactosamine and methyl- α-galactoside) or LecB (methyl-α-fucoside) at concentrations of 15–50 mM, but not glucose, reduced the negative effects caused by the injection of bacteria and bacterial dissemination in the lungs and blood. Furthermore, methyl-α-galactose and N-acetylgalactosamine led to a better survival rate among the infected mice. The effectiveness of synthetic lectins inhibitors, tetravalent galactosylated, and fucosylated calixarenebased glycoclusters (*Table*, compounds 5 and 6) was studied in the same model of acute lung infection in mice [40]. As might have been expected, glycoclusters showed much higher effectiveness both in retaining the lung barrier function and in reducing the bacterial dissemination in the lungs and spleen compared to monosaccharides at same concentrations (1–5 mM). Lung permeability for labeled albumin after glycoclusters had been added was the same as after the injection of strains with mutations in the lectin genes [39]; the bacterial dissemination in lungs and spleen decreased by 1–3 orders of magnitude. The fucosylated glycocluster was more effective. Unfortunately, no data on the survival rate were available.

Several cases in which saccharide solutions were used to treat a *P. aeruginosa *infection in humans have also been reported. Steuer *et al*. [103] demonstrated the effectiveness of using *D*-galacose, *D*-mannose, and sialic acid solutions to treat external otitis caused by infection with *P. aeruginosa*, although in this case the effect could have been due to inhibition of some other adhesins besides lectins. A case of successful treatment of the upper airway infection in a child subjected to chemotherapy has also been reported [104]. The infection was resistant to antibiotics, while inhalation of galactose and fucose solutions resulted in the complete elimination of the pathogen. Finally, Hauber *et al*. studied inhalation of solutions of these saccharides to treat cystic fibrosis patients whose lungs were chronically colonized by *P. aeruginosa *[105]. A twice-daily inhalation of the saccharide solution for 21 days significantly reduced bacterial counts in the patients’ sputum and inhibited TNFα expression. Unfortunately, no statistically significant improvement in lung function was observed, which was related to the insufficient sample size.

Although the effect of using monosaccharides in the aforementioned studies was often rather small, recent data have demonstrated that these limitations can be potentially overcome by using multivalent compounds. The results of these studies have convincingly confirmed that positive results can be achieved *in vivo *by inhibition of *P. aeruginosa *lectins.

## CONCLUSIONS

Lectins LecA and LecB seem to be among the virulence factors of *P. aeruginosa*: they contribute to the ability of this organism to colonize human tissues and organs and persist in them as biofilms, thus causing hard-totreat chronic diseases. Both lectins affect bacteria’s ability to attach to epithelial human cells, are the key components of bacterial biofilms, and can inhibit the ciliary movement and disturb the barrier function of epithelial tissue. Unfortunately, it remains unclear how important each of these lectin functions in *in vivo *infection is. Taking into account the fact that lectin specificity differs, it cannot be ruled out that *P. aeruginosa* LecA and LecB play different roles in the infection of a human organism. However, regardless of the nature of lectins function, the use of corresponding monosaccharides and multivalent glycoclusters in animal models of a *P. aeruginosa *infection has reliably demonstrated the positive effect of the inhibition of both lectins, which has also been confirmed by clinical data.

Among the variety of lectin inhibitors, the most promising ones are those where the multivalence effect is used to achieve a higher affinity to their targets. This is partially related to the multivalence of lectins: compounds bearing several affine groups can simultaneously bind two monomers from a single tetramer. The molecular modeling method has demonstrated that some compounds potentially possess this ability. It was found that some classes of multivalent compounds can inhibit the development of *P. aeruginosa *biofilms and/ or impede bacterial adhesion to epithelial cells. The positive effect of tetravalent calixarene-based glycoclusters in an *in vivo *acute model of lung infection was also demonstrated.

Most multivalent lectin inhibitors are synthetic glycodendrimers and glycoclusters. The cost intensity and complexity of synthesizing many of them can be a significant obstacle in further advancement towards preclinical and especially clinical trials. Furthermore, there is a high risk of adverse toxic effects and non-optimal pharmacokinetic properties. In this regard, natural neutral polysaccharides, such as the galactomannan or oligosaccharides produced by their hydrolysis, seem to have a higher potential. Plant-derived galactomannans are widely used in the food industry, are safe, and exceptionally inexpensive. However, the effectiveness of natural polysaccharides, as well as most synthetic glycoclusters and glycodendrimers, is yet to be confirmed using animal models of infection. We believe that, taking into account the encouraging results achieved in experiments with calixarenes, the highest priority objective is to further verify the effectiveness of natural and synthetic multivalent compounds (and probably their combinations with conventional antibiotics) *in vivo *in various models of infection.

## Abbreviations

IPTG: isopropyl-β-D-thiogalactopyranoside
PQS: Pseudomonas quinolone signal
IFN-γ: interferon-γ
Lea, Lex: Lewis oligosaccharides
TNFα: tumor necrosis factor α
ITC: isothermal titration calorimetry
ELLA: enzyme-linked lectin assay
SPR: surface plasmon resonance
